# PI3Kδ Regulates the Magnitude of CD8^+^ T Cell Responses after Challenge with *Listeria monocytogenes*

**DOI:** 10.4049/jimmunol.1501227

**Published:** 2015-08-26

**Authors:** Verity Q. Pearce, Hicham Bouabe, Amy R. MacQueen, Valentina Carbonaro, Klaus Okkenhaug

**Affiliations:** Laboratory of Lymphocyte Signalling and Development, Babraham Institute, Cambridge CB22 3AT, United Kingdom

## Abstract

PI3Ks regulate diverse immune cell functions by transmitting intracellular signals from Ag, costimulatory receptors, and cytokine receptors to control cell division, differentiation, survival, and migration. In this study, we report the effect of inhibiting the p110δ subunit of PI3Kδ on CD8^+^ T cell responses to infection with the intracellular bacteria *Listeria monocytogenes*. A strong dependency on PI3Kδ for IFN-γ production by CD8^+^ T cells in vitro was not recapitulated after *Listeria* infection in vivo. Inactivation of PI3Kδ resulted in enhanced bacterial elimination by the innate immune system. However, the magnitudes of the primary and secondary CD8**^+^** T cell responses were reduced. Moreover, PI3Kδ activity was required for CD8^+^ T cells to provide help to other responding CD8^+^ cells. These findings identify PI3Kδ as a key regulator of CD8^+^ T cell responses that integrates extrinsic cues, including those from other responding cells, to determine the collective behavior of CD8^+^ T cell populations responding to infection.

## Introduction

CD8^+^ T cells respond to Ags presented by MHC class I molecules and provide protective immunity against intracellular pathogens and cancer. Following infection, low frequencies of naive CD8^+^ T cells whose TCRs recognize specific peptide Ags undergo massive proliferative expansion during which responding cells differentiate to become CTLs (1, 2). CTLs kill target cells, often leading to elimination of pathogens, after which most CTLs die to restore immune homeostasis. However, a small proportion of CTLs survive following primary infection and establish themselves in the lymph nodes and bone marrow as long-term memory T cells (Tmem) (1–3). Robust recall responses by Tmem contribute to enhanced protection upon pathogen re-encounter (1–3). Thus, dynamic regulation of the size and function of CD8^+^ T cell populations responding to infection is required for appropriate control of immunity.

In CD8^+^ T cells, the PI3K pathway is activated by TCR, cytokine receptor, and costimulatory signaling (4). Despite this, the function of the PI3K pathway in regulating primary and secondary immune responses to infections is poorly elucidated. The class I PI3Ks, of which there are four types (PI3Kα, PI3Kβ, PI3Kδ, and PI3Kγ), are a subfamily of evolutionarily conserved kinases that catalyze the phosphorylation of phosphatidylinositol(4,5)-biphosphate to generate the second messenger signaling molecule phosphatidylinositol(3,4,5)-trisphosphate [PtdIns(3,4,5)P_3_] (5). PI3Kδ (which is a heterodimer of a p110δ catalytic subunit in complex with an SH2-containing regulatory subunit p85α or p85β) is predominantly responsible for activating the Akt signaling pathway in CD8^+^ T cells stimulated through the TCR and/or IL-2 receptor (6–9). PI3Kγ (which is a heterodimer of p110γ and a p101 or p84 regulatory subunit) is primarily responsible for signaling via some inflammation-induced chemokine receptors in activated CD8^+^ T cells, but it can also make a minor contribution to TCR-induced Akt activation in naive T cells (10–13). In comparison with PI3Kδ and PI3Kγ, the contribution of PI3Kα and PI3Kβ to PI3K signaling in T cells is thought to be minimal (4, 5, 14). PtdIns(3,4,5)P_3_ acts as a tether for intracellular proteins with pleckstrin homology domains on the cytosolic surface of the lipid bilayer. Key among these are the serine-threonine kinases Pdk1 and Akt. The activity of Akt is regulated by phosphorylation by Pdk1 at Thr^308^, an event that is dependent on PtdIns(3,4,5)P_3_ binding to the pleckstrin homology domains of both Akt and Pdk1 (15–17). A major role for Akt is to phosphorylate transcription factors (TFs) of the Foxo family, sequestering them in the cytosol where they are degraded (5, 18). Consequently PI3K signaling can negatively regulate Foxo TF target genes, such as *Klf-2*, *Sell, Ccr7, Il7r*, and *S1p* (8, 9, 19–21). By suppressing the expression of these Foxo target genes, PI3K can help T cells prepare for lymph node exit and the initiation of an immune response in the peripheral tissues. To what extent the failure to regulate these trafficking events affects systemic immune responses remains to be fully elucidated.

Foxo TFs have also been proposed to differentially regulate the expression of the transcription factors T-bet and eomesodermin (Eomes), favoring the formation of CD8^+^ Tmem by repressing the expression of T-bet and promoting that of Eomes (22). Similarly, the inhibition of mammalian target of rapamycin (mTOR) has been shown to favor the formation of CD8^+^ Tmem via differential regulation of T-bet and Eomes (23, 24). The relationship between PI3K and mTOR is complex (5). Although PI3K and Akt contribute to mTOR activity in some cell systems, in CD8^+^ T cells other signaling pathways may contribute more strongly to mTOR activation (7, 8, 25, 26). In turn, mTOR can phosphorylate Akt at Ser^473^ in a PI3K-independent fashion (22). It is therefore important to appreciate that the PI3Ks regulate signaling proteins in addition to Akt, mTOR, and Foxo TFs and, moreover, that mTOR can be controlled by signaling pathways other than PI3K (5).

The contribution of PI3Kδ to T cell development and function has been studied using p110δ^D910A^ mice (in which the p110δ subunit of PI3Kδ is inactivated by a point mutation in the catalytic domain), p110δ^−/−^ mice, p85-deficient mice, and/or small molecule inhibitors such as IC87114 that selectively inhibit PI3Kδ (5, 27–29). We have found that CD8^+^ T cells from p110δ^D910A^ mice, or wild-type (WT) T cells stimulated in the presence of IC87114, respond poorly to stimulation with Ag in vitro (6). In particular, IFN-γ production was profoundly diminished in p110δ^D910A^ CD8^+^ T cells and could be suppressed in WT T cells using very low concentrations of IC87114 (6). We and others have also found that the production of cytotoxic proteins produced in CTL granules such as granzyme B (GzmB) and perforin were impaired in vitro, albeit to different extents, when PI3Kδ was inhibited (30, 31). However, mice that lack both the p85α and p85β regulatory subunits in T cells respond normally to infection with mouse hepatitis virus despite impaired PI3K signaling (32). Moreover, we have recently shown that tumors are killed more effectively by the immune system in p110δ^D910A^ mice than in WT mice (33). Enhanced killing of tumors was the consequence of inhibition of p110δ within regulatory T cells, which helped unleash more potent CD8^+^ T cell–dependent antitumor responses (33). These findings imply that CD8^+^ T cell function is not entirely abrogated with inactivation of PI3K p110δ and compelled us to further investigate the function of PI3K signaling in CD8^+^ T cell responses in vivo.

In this study, we have examined the contribution of PI3Kδ to CD8^+^ T cell responses to infection with *Listeria monocytogenes. L. monocytogenes* is a Gram^+^ intracellular bacteria widely used to study the dynamics of CD8^+^ T cell responses to infection (34). We show that the magnitude of both primary and secondary CD8^+^ T cell responses to *L. monocytogenes* infection is reduced in p110δ^D910A^ mice. The altered response was attributable to lack of PI3Kδ activity both intrinsic and extrinsic to responding CD8^+^ T cells. Cotransfer studies demonstrated the ability of WT CD8^+^ T cells to provide help to p110δ^D910A^ T cells, suggesting a role for PI3Kδ in mediating signals required for CD8^+^ T cells to support the proliferation of other responding CD8^+^ T cells. Unexpectedly, after challenge with *L. monocytogenes*, p110δ^D910A^ CD8^+^ T cells produced normal or enhanced levels of IFN-γ, IL-2, and GzmB. Moreover, p110δ^D910A^ mice generated normal or enhanced numbers of long-lived memory T cells and raised a robust recall response a year after initial challenge. These findings identify PI3Kδ as a key regulator of CD8^+^ T cell responses that integrates extrinsic cues, including from other responding cells, to determine the collective behavior of CD8^+^ T cell populations responding to infection.

## Materials and Methods

### Chemicals

Chemicals were ordered from Sigma-Aldrich unless otherwise specified. IC87114 was synthesized by Jonathan Clark (Babraham Institute), as described previously (6).

### Bacteria

*L. monocytogenes*–expressing OVA (Lm*-*OVA) (35, 36) and its attenuated derivative (*ΔactA* Lm-OVA) (37) were obtained from Dr. Hao Shen (University of Pennsylvania) or purchased (DMX Bio). Mice were infected i.v. with 1 × 10^4^ CFU Lm-OVA or 1 × 10^6^ *ΔactA* Lm-OVA unless otherwise stated. Frozen stocks were thawed at room temperature, diluted in PBS, and injected within 2 h. Actual CFU injected was routinely verified by plating out the stocks that had been thawed for injection.

### Mice

The following mouse strains used in this study have been described previously and had been backcrossed to the C57BL/6 background for ≥10 generations: p110δ^D910A^ (MGI:2385596), OT1 (MGI:3054907), YETI (MGI:3665254) (6, 38–40). All experimental protocols were approved by the Babraham Institute Animal Welfare and Experiment Committee and the Home Office (PPL 80/2248 and 70/7661).

### CD8^+^ T cell isolation

Lymphocyte suspensions were incubated for 30 min at 4°C with 2.5 μg/ml FITC-conjugated anti-CD4α, anti-B220, anti-CD25, anti-CD69, anti-CD49b, and anti–MHC class II Abs. Next, the cells were washed and incubated for 20 min at 4°C with 100 μl anti-FITC MACS magnetic beads (Miltenyi Biotec) per 10^8^ cells. Cells were washed again and then applied to a MACS LS column (Miltenyi Biotec), and the flow-through was collected. The resulting cells were >90% CD8^+^, as assessed by flow cytometry.

### Biochemistry

CD8^+^ T cells (3 × 10^6^) were incubated with biotinylated Abs against CD3 (145-2C11, 1 μg/ml), anti-CD8 (53-6.7, 10 μg/ml), and anti-CD28 (37.51, 2 μg/ml) (all from BioLegend) on ice for 30 min in 85 μl RPMI 1640 with 0.5% FCS. Cells were warmed at 37°C for 2 min before adding 85 μl 1:50 streptavidin (Jackson ImmunoResearch Laboratories, 016-000-084) and incubating for 5 min at 37°C. The cells were pelleted by centrifugation and resuspended in 50 μl ice-cold lysis buffer (50 mM HEPES, 150 mM NaCl, 10 mM NaF, 10 mM NaF, 10 mM indoacetamide, 5 μl protease inhibitor [Proteoloc], 5 μl EDTA [Proteoloc], 1% [w/v] Nonidet P-40 [BDH]) and incubated for 10 min on ice.

Lysates were centrifuged at 14,800 rpm at 4°C in a microcentrifuge. The supernatant was subsequently mixed with 16.7 μl NuPAGE LDS sample buffer (Life Technologies) and frozen. Lysates were thawed at room temperature and 1 μl 1 M DTT (Life Technologies) was added. The proteins were separated on a 10% Bis-Tris gel (Life Technologies) and transferred to polyvinylidene difluoride membranes that were probed with the indicated Abs (all from eBioscience except anti–β-actin, which was from Santa Cruz Biotechnology.

### Flow cytometry

The following Abs were from eBioscience unless otherwise noted: anti-CD8a (53-6.7), anti-CD62L (MEL-14, Caltag Laboratories), anti-CD44 (IM7), anti-CD4 (GK1.5 or RM4-5, BD Biosciences), anti-CD127 (Lg.7F9), anti-CD45.1 (A20), anti-CD45.2 (104), anti-KLRG1 (2F1), anti-CCR7 (4B12), anti-CD183 (CXCR3-173, BioLegend), anti-Eomes (Dan11mag), anti–IFN-γ (XMG1.2), anti–T-bet (eBio4B10), and anti-GzmB (GB12, Invitrogen). The iTAg K^b^ MHC tetramer loaded with SIINFEKL peptide (Tet) was purchased from Beckman Coulter or MBL International. Blood cells were stained undiluted at room temperature for 30 min. Red cells were lysed using RBC lysing buffer (Sigma-Aldrich) and then the cells were washed with FACS buffer (PBS containing 2% FCS, 0.1% NaN_3_). Cells from the spleen, bone marrow, and lymph nodes were stained in FACS buffer. Samples were acquired using a BD LSR II flow cytometer. Data were analyzed using FlowJo software (Tree Star, Ashland, OR). For cytokine and GzmB detection, 10^6^ lymphocytes from splenic single-cell suspensions of mice that had received 500 OT1 T cells were restimulated with 10 μM OVA_257–264_ peptide for 5 h, the last 3 h in the presence of brefeldin A (eBioscience). After incubation with surface Abs for 30 min on ice, cells were washed and fixed with IC fixation buffer (eBioscience). The cells were then permeabilized with permeabilization wash buffer (BioLegend) and incubated with intracellular Abs against intracellular proteins IFN-γ (XMG1.2), IL-2 (JES6-SH4), or GzmB (16G6) for 30 min on ice before washing and analysis. The gating strategy was as follows: gate 1, lymphocyte population; gate 2, CD8 versus Tet; gate 3, CD45.1 versus CD45.2 (if applicable). Samples were spiked with Flow-Count fluorospheres (Beckman Coulter), which were used to calculate cell numbers in each sample.

### In vitro stimulation of T cells

Stimulation of T cells in vitro was carried out by stimulating 2 × 10^5^ cells/well of total lymph node cell suspension with 1 μg/ml anti-CD3 (145-2C11), 1:500 dilution of anti-CD28 (37.51) hybridoma supernatant, 3 ng/ml IL-12 in media (RPMI 1640 (Life Technologies, no. 21875-034) plus 5% heat-inactivated FCS (LabTech) plus 1% penicillin/streptomycin (Invitrogen, 15070-063) plus 50 μM 2-ME (Sigma-Aldrich, M3701) in a 96-well plate (Nunc). For the last 90 min of each time point, cultures were incubated with 10 μg/ml frefeldin A (eBioscience, 15406-51) at 37°C then stained for surface markers, fixed, and permeabilized as previously described before analysis on an LSRFortessa flow cytometer.

### Adoptive cell transfers

OVA-specific CD8^+^ T cells obtained from the lymph nodes of WT OT1 or p110δ^D910A^ OT1 mice and purified by magnetic sorting as described above. The number of OT1 cells was calculated based on purity determined by flow cytometry, and indicated numbers or OT1 cells (usually 500) were injected i.v. into the lateral tail vein of mice 18 h prior to infection with Lm-OVA.

### 5-Ethynyl-2′-deoxyuridine labeling of lymphocytes in vivo

WT and p110δ^D910A^ mice were infected i.v. with ∼5 × 10^6^ CFU *ΔactA* Lm-OVA. 5-Ethynyl-2′-deoxyuridine (EdU, 250 μg; Life Technologies), dissolved in PBS, was injected i.v. 24 h before culling the mice and collection of organs. Splenocytes were then subjected to cell surface immunostaining, and the incorporation of EdU by DNA was detected by intracellular fluorogenic “click” reaction using a Click-iT Plus EdU Alexa Fluor 647 flow cytometry assay kit (Life Technologies), according to the manufacturer’s protocol. Briefly, fixed and permeabilized cells were incubated with copper and Alexa Fluor 647 dye–labeled picolyl azide at room temperature for 30–45 min. Copper catalyzes a covalent reaction between picolyl azide and alkyne found in the ethynyl moiety of EdU.

### Detection of apoptosis

Splenocytes isolated from WT and p110δD910A mice 5 d after i.v. infection with ∼5 × 106 CFU *ΔactA* Lm-OVA were incubated (at ∼3–5 × 10^6^ cells/well in a 96-well plate) in RPMI 1640, 5% FCS, antibiotics, and 0.75 μM FAM–l-valyl–l-alanyl–l-aspartic acid(methyl ester)–fluoromethyl ketone (FAM-VAD-FMK, Intracellular Technologies) for 3 h at 37°C. Cells were then immunostained and analyzed by a flow cytometer. Dead/necrotic cells (positive for viability dye) were gated out and the percentage of Tet^+^CD8^+^ cells labeled with FAM-VAD-FMK was determined.

## Results

### PI3Kδ is required for Akt phosphorylation and IFN-γ production by p110δ^D910A^ T cells stimulated in vitro

**FIGURE 1.**
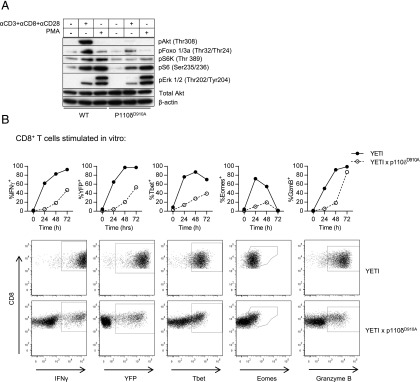
Inhibition of PI3Kδ leads to defective signaling and CD8^+^ T cell differentiation in vitro. PI3Kδ is essential for Akt and Foxo phosphorylation in CD8^+^ cells. Serine–threonine kinase signaling in p110δ^D910A^ CD8^+^ T cells. (**A**) Purified CD8^+^ T cells from WT and p110δ^D910A^ mice were stimulated with biotinylated Abs against CD3, CD8, and CD28 and crosslinked with streptavidin. Western blots of the cell lysates were probed with the indicated phospho-specific Abs. Abs against Akt and β-actin were used as loading controls. Experiment is representative of two repeats. (**B**) Expression of IFN-γ, YFP, T-bet, Eomes, and GzmB by YETI or p110δ^D910A^ YETI CD8^+^ T cells activated with anti-CD3, anti-CD28, and IL-12, as determined by flow cytometry. The plots are representative of CD8^+^ T cells 48 h after stimulation. Similar results were obtained in three independent experiments.

To test the role of PI3Kδ in transducing signals downstream of the TCR and CD28, we stimulated CD8^+^ T cells purified from WT and p110δ^D910A^ mice and monitored phosphorylation of key signaling proteins. Phosphorylation of Akt, Foxo TFs, and p70S6K was profoundly diminished in p110δ^D910A^ CD8^+^ T cells (Fig. 1A), indicating that p110δ is the predominant PI3K isoform contributing to Akt phosphorylation in CD8^+^ T cells. However, some compensatory signaling was evident, because phosphorylation of Foxo, S6, and Erk was detected in p110δ^D910A^ T cells. Moreover, assessment of the response to stimulation by PMA demonstrated that whereas the S6K and Erk pathways can be rescued by diacylglycerol-dependent signaling pathways in p110δ^D910A^ cells, Akt and Foxo phosphorylation cannot. Hence, compensatory activation of the Ras-Erk pathway can contribute to mTOR activity independently of PI3K and Akt, consistent with previous results (7, 26). T cells from YETI mice express yellow fluorescent protein (YFP) when the *Ifng* gene is actively being transcribed (39). To examine the role of PI3Kδ in promoting IFN-γ production more closely, we used cells from YETI mice to monitor the proportion of CD8^+^ T cells able to produce IFN-γ after stimulation with anti-CD3, anti-CD28, and IL-12. We found that PI3Kδ inactivation reduced YFP and IFN-γ expression in CD8^+^ T cells (Fig. 1B). These experiments also showed that during in vitro activation of CD8^+^ T cells, T-bet, Eomes, and GzmB expression were lower in p110δ^D910A^ T cells (Fig. 1B).

### PI3Kδ regulates the magnitude of primary and secondary responses to *L. monocytogenes* infection

**FIGURE 2.**
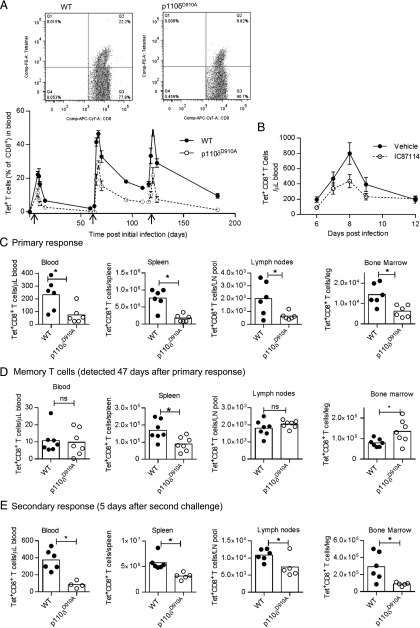
Defective primary and secondary CD8^+^ T cell responses in p110δ^D910A^ mice. (**A**) WT or p110δ^D910A^ mice were infected with Lm-OVA on days 0, 62, and 115 (indicated with arrows) with 10^4^ CFU Lm-OVA. The graph shows the percentage of CD8^+^ T cells in blood samples that were Tet^+^; *n* = 5 or 6 per group. Statistical analyses: two-way ANOVA of days 0–15, *p* = 0.002; days 63–110, *p* < 0.001; days 116–183, *p* = 0.0013. Results are representative of two independent experiments. (**B**) Mice treated with IC87114 via the food from days −1 to 10 and infected on day 0 with Lm-OVA. The number of Tet^+^CD8^+^ T cells in the blood were enumerated by flow cytometry; *n* = 8/group. Statistical analyses: two-way ANOVA, *p* = 0.049. Results are representative of two independent experiments. (**C**–**E**) Number of Tet^+^CD8^+^ T cells recovered from blood (cells per μl), spleen (cells per spleen), lymph nodes (total from six peripheral lymph nodes), or bone marrow (cells from one femur and one tibia). Cells were harvested 8 d after initial infection (C), 47 d after initial infection (D), or 5 d after second challenge (day 58) with Lm-OVA; *n* = 4–6 per group. *p < 0.05 by unpaired *t* test. Results are representative of two (A–D) or three (E) independent experiments.

To track CD8^+^ T cells responding to infection, we infected mice with a transgenic strain of *L. monocytogenes* that expresses chicken OVA and detected OVA-specific CD8^+^ T cells using MHC class I H-2^b^ tetramers loaded with OVA_257–264_ (SIINFEKL) peptide. The proportions of CD8^+^ T cells in the blood that stained positive with the tetramer (Tet^+^ CD8^+^) were reduced in p110δ^D910A^ mice (Fig. 2A). This reduction in the proportion of Tet^+^CD8^+^ T cells was apparent both at the peak of response as well as among the Tmem that persisted and could be detected before and after subsequent challenges (Fig. 2A). Nevertheless, the p110δ^D910A^ mice did show evidence of CD8^+^ Tmem formation, as their secondary responses were more rapid and achieved greater peak magnitude than during their primary response (Fig. 2A). Diminished CD8^+^ T cell responses could either be due to a developmental defect that occurs as a result of a congenital lack of p110δ activity in p110δ^D910A^ mice, or due to an acute requirement for PI3Kδ activity during T cell activation in vivo. To distinguish between these, we treated WT mice with IC87114 and infected them with Lm-OVA. IC87114 caused a similar defect in the primary response to that seen with the p110δ^D910A^ mice, demonstrating that acute PI3Kδ activity is required for appropriate CD8^+^ T cell response kinetics (Fig. 2B).

PI3Kδ affects the expression of various receptors, including CD62L and CCR7, which facilitate trafficking of T cells to the lymph nodes (8, 9). Consistent with this, p110δ^D910A^ Tet^+^CD8^+^ T cells in the lymph nodes still expressed high levels of CD62L 8 d postinfection (Supplemental Fig. 1). Nevertheless, most p110δ^D910A^ Tet^+^CD8^+^ T cells in the spleen and blood effectively downregulated the expression of CD62L (Supplemental Fig. 1), indicating partially redundant pathways for regulation of lymphoid-homing molecules in CD8^+^ T cells.

To address whether the reduced proportion of T cells in the blood reflected their altered distribution, we enumerated Tet^+^CD8^+^ T cells in the spleen, lymph nodes, and bone marrow (Fig. 2C–E). In p110δ^D910A^ mice the numbers of Tet^+^CD8^+^ T cells were reduced at the peak of the primary response (8 d postinfection) in all organs examined (Fig. 2C). However, the number of Tet^+^CD8^+^ T cells detected 47 d postinfection were reduced in the spleen, similar in the blood and lymph nodes, but increased in the bone marrow (Fig. 2D). During secondary responses (5 d after second challenge), Tet^+^CD8^+^ T cell numbers were again reduced in all tissues examined (Fig. 2E). These data indicate that p110δ^D910A^ cells produce near normal numbers of Tmem, but that the p110δ^D910A^ Tmem, similar to their naive precursors, produce an effector T cell (Teff) response of reduced magnitude upon acute challenge with Lm-OVA.

### p110δ^D910A^ mice generate normal numbers of memory precursor effector cells in the lymph nodes and bone marrow and mount intact long-term memory T cell responses

**FIGURE 3.**
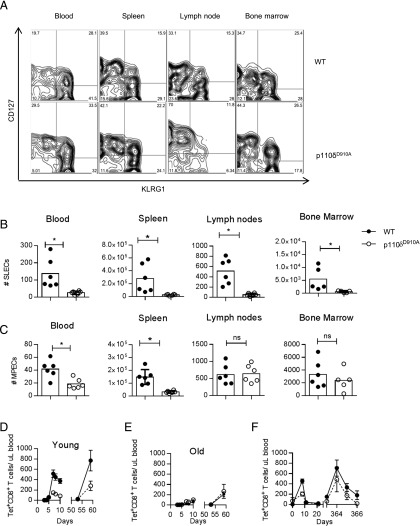
Intact formation of memory T cells and long-term memory responses in p110δ^D910A^ mice. Tet^+^CD8^+^ T cells in blood, spleen, lymph nodes, and bone marrow 8 d postinfection with Lm-OVA are shown. (**A**) Representative FACS profiles showing CD127 and KLRG1 expression on WT or p110δ^D910A^ Tet^+^CD8^+^ T cells isolated from the indicated organs 8 d after primary infection with Lm*-*OVA. (**B** and **C**) Graphical representation of CD127^high^KLRG1^low^ MPECs (B) and CD127^low^KLRG1^high^ SLECs (C) 8 d post infection. *n* = 6, representative experiment of three. *p < 0.05 by unpaired *t* test. (**D** and **E**) WT or p110δ^D910A^ mice were infected with Lm-OVA on days 0 and 54 and the number of Tet^+^CD8^+^ T cells in the blood were enumerated by flow cytometry. (D) Mice were 9 wk old at the start of experiment. (E) Mice were 70 wk at the start of the experiment. (**F**) WT or p110δ^D910A^ mice were infected with Lm-OVA on days 0 and 359 and the number of Tet^+^CD8^+^ T cells in the blood was enumerated at regular intervals by flow cytometry. *n* = 5-6/group. Statistical analyses: two-way ANOVA, days 0–20, *p* = 0.041; days 363–366, not significantly different. Results are representative of two independent experiments.

During the primary immune response, responding CD8^+^ T cells can be classified as short-lived effector cells (SLECs) or as memory precursor effector cells (MPECs) based on the differential expression of KLRG1 and CD127 (41). SLECs (KLRG1^high^, CD127^low^) are responsible for the immediate clearance of bacteria and most will die following resolution of the infection. Some of the MPECs (KLRG1^low^, CD127^high^), in contrast, will form long-lived memory T cells. In p110δ^D910A^ mice, we found a significant reduction in SLECs in all tissues analyzed 8 d following infection (Fig. 3A, 3B). There were also fewer MPECs in the blood and spleen of p110δ^D910A^ mice (Fig. 3A, 3C). However, the numbers of MPECs in the lymph nodes and bone marrow were similar in WT and p110δ^D910A^ mice (Fig. 3A, 3C). Because the lymph nodes and bone marrow are thought to be the primary site for the generation and maintenance of memory T cells (3, 42), these results further support the notion that PI3Kδ inhibition preferentially impairs the generation of effector cells without having a strong impact on the generation of Tmem.

### Long-term memory responses and aging-related loss of CD8^+^ T cell function

Memory T cell responses can provide life-long protection against specific Ags (3). With the aim to examine the role of PI3Kδ in the long-term persistence and function of memory CD8^+^ T cells, we started by comparing immune responses in young and old mice. Mice can live for 2–3 y, but mortality from age-related decline increases in frequency from ∼18 mo of age (43). We therefore compare immune responses in mice that were 2–4 mo of age (young) to mice that were 15–18 mo of age (old). The old mice mounted reduced primary and secondary immune responses when compared with young mice (compare Fig. 3D and 3E). However, old p110δ^D910A^ mice mounted primary and secondary CD8^+^ T cell responses that were similar in magnitude to old WT mice (Fig. 3E). Moreover, both WT and p110δ^D910A^ mice that had been infected when young and then reinfected a year later mounted recall responses of similar magnitudes despite the impaired primary response observed in young p110δ^D910A^ mice (Fig. 3F). Hence, the impaired primary and secondary CD8^+^ T cell responses in young p110δ^D910A^ mice give rise to memory T cells that persist into old age to elicit recall responses that are similar in magnitude to those raised by old WT mice.

### PI3Kδ activity within CD8^+^ T cell populations is required for optimal expansion

**FIGURE 4.**
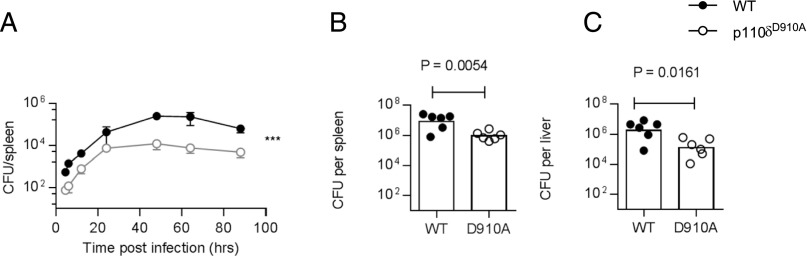
Enhanced bacterial clearance by p110δ^D910A^ mice. (**A**) WT or p110δ^D910A^ mice were infected with Lm-OVA CFUs recovered from the spleen enumerated at indicated times after infection; *n* = 3–4/group, bars show SEM. Statistical analyses: *p* = 0.0002 by two-way ANOVA. (**B** and **C**) Number of CFUs recovered from the spleen (B) or liver (C) 72 h postinfection from a separate experiment from (A) after injection of 4 × 10^4^ CFU Lm-OVA. Similar results were obtained from four similar experiments.

The reduced primary and recall T cell response in young mice could be a consequence of impaired signaling within CD8^+^ T cells or due to factors extrinsic to the responding T cells, such as differences in bacterial load, Ag presentation, or cytokine production by macrophages and dendritic cells and/or paracrine help from other responding or bystander T cell populations. We recovered reduced numbers of *L. monocytogenes* colonies from the spleen of p110δ^D910A^ mice compared with WT mice during the first 4 d postinfection (Fig. 4A). Because p110δ^D910A^ spleens are smaller than WT spleens, we also measured CFU in the liver and found again the counts to be significantly lower in p110δ^D910A^ hosts than in WT hosts, similar to what we found in the spleen (Fig. 4B, 4C). Because *Listeria* clearance happens before *Listeria*-specific CD8^+^ T cells can be detected, these findings suggest that PI3Kδ inhibition can enhance bacterial clearance by the innate immune system.

**FIGURE 5.**
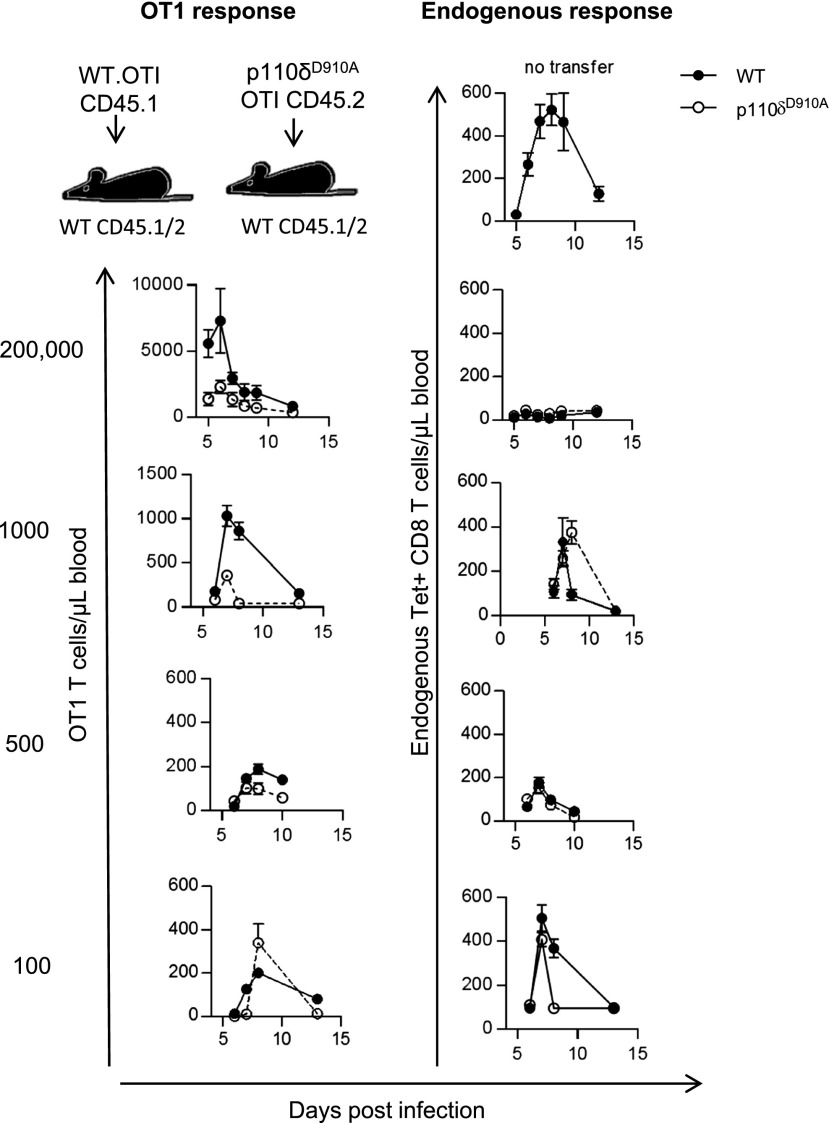
CD8 T cell–intrinsic role for p110δ is dose-dependent. The indicated number of purified WT OT1 T cells (CD45.1^+^) or p110δ^D910A^ OT1 T cells (CD45.2^+^) were transferred into WT recipient mice (CD45.1^+^CD45.2^+^), which were infected 1 d later with Lm-OVA and the number of OT1 T cells and endogenous Tet^+^CD8^+^ CD8 T cells in the blood was determined by flow cytometry. The *left panel* shows number of transferred OT1 T cells recovered from blood samples (filled symbols indicate WT OT1; empty symbols indicate p110δ^D910A^ OT1). The *right panel* shows endogenous Tet^+^CD8^+^ T cells of WT mice receiving WT OT1 T cells (filled symbols) or p110δ^D910A^ OT1 T cells (empty symbols); *n* = 4–8/group. Results are representative of two to four independent experiments.

We therefore sought to determine whether PI3Kδ plays a cell-intrinsic role in regulating the expansion of CD8^+^ T cells following infection in the presence of WT innate immune cells. We started by injecting different numbers of CD45.1^+^ WT or CD45.2^+^ p110δ^D910A^ Tet^+^CD8^+^ T cells expressing the OT1 TCR transgene into WT recipient mice expressing CD45.2 and CD45.1 Ags before infecting recipient mice with Lm-OVA. When 200,000, 1,000, or 500 OT1 T cells were injected, the magnitude of expansion by p110δ^D910A^ OT1 T cells was reduced compared with WT OT1 T cells (Fig. 5, *left panel*). Interestingly, however, when 100 OT1 T cells were injected¸ the difference between the expansion of WT and p110δ^D910A^ T cell responses was less noticeable (Fig. 5, *left panel*), indicating that the function of PI3Kδ is dependent on the size of the responding population.

Consistent with previously published results (44), transfer of 200,000 OT1 T cells quenched endogenous T cell responses and this was similar for both genotypes, indicating that the p110δ^D910A^ T cells could compete effectively with endogenous WT T cells for access to APCs (Fig. 5, *right panel*). When 500 OT1 T cells were injected, the magnitude of the donor and recipient Tet^+^CD8^+^ T cell responses were similar. Hence, to mimic physiological starting populations we injected 500 donor OT1 T cells per recipient before infection with Lm-OVA in subsequent experiments.

**FIGURE 6.**
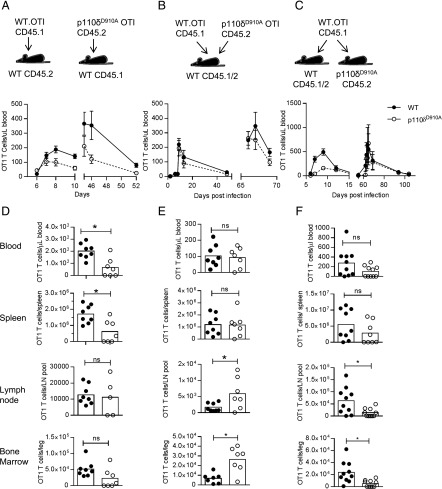
T cell–intrinsic and –extrinsic roles for p110δ during primary and secondary responses. (**A**) Five hundred WT (CD45.1) or p110δ^D901A^ (CD45.2) OT1 T cells were transferred into WT hosts (CD45.1 or CD45.2) on day −1. Mice were then infected with Lm-OVA on days 0 and 40. Graphs show the number of OT1 T cells enumerated in the blood following primary and secondary infection by flow cytometry; *n* = 7–8/group. Filled symbols indicate WT OT1; empty symbols indicate p110δ^D910A^ OT1 T cells. Statistical analyses: two-way ANOVA, days 6–10, *p* = 0.0415; days 45–25, *p* = 0.0409. Results are representative of three independent experiments. (**B**) Two hundred fifty WT (CD45.1) and 250 p110δ^D901A^ (CD45.2) OT1 T cells were cotransferred into WT hosts (CD45.1/CD45.2) and infected with Lm-OVA the next day and 62 d later. The number of WT and p110δ^D910A^ OT1 T cells in the blood is shown. Filled symbols indicate WT OT1 T cells; empty symbols indicate p110δ^D910A^ OT1 T cells; *n* = 8/group. Statistical analyses: two-way ANOVA, days 2–43 and days 66–69 genotypes, not significantly different. Results are representative of three independent experiments. (**C**) Five hundred WT OT1 T cells (CD45.1) were transferred to either WT (CD45.1/CD45/2) or p110δ^D910A^ (CD45.2) mice and infected the following day and then again on day 60 with Lm-OVA. The number of WT OT1 T cells in blood is shown. Filled symbols indicate OT1 cells in WT hosts; empty symbols indicate OT1 cells in p110δ^D910A^ hosts; *n* = 8/group. Statistical analyses: day 1–12, *p* = 0.0061; days 62–94, not statistically different (two-way ANOVA). (**D**–**F**) Number of donor OT1 cells recovered from indicated organs at the peak of the secondary or tertiary responses in repeat experiments, corresponding to (A)–(C). (D) Mice were infected on days 0, 43, and 111 and the number of OT1 T cells in the blood, spleen lymph nodes, and bone marrow was enumerated by flow cytometry at peak tertiary response (day 5 following third infection); *n* = 7–8/group. Results are representative of two independent experiments. (E) Mice were infected days 0 and 68 and the number of OT1 T cells in the blood, spleen, lymph node, and bone marrow was enumerated by flow cytometry at peak secondary response (day 5 following second infection); *n* = 7–8/group. Results are representative of two independent experiments. (F) Mice were infected on days 0 and 52 and the number of WT OT1 T cells in the blood, spleen, lymph nodes, and bone marrow was enumerated by flow cytometry at peak secondary response (day 5 after second infection); *n* = 8/group. Results are representative of three independent experiments. *p < 0.05 by Student unpaired *t* test.

To further examine the T cell–intrinsic role for PI3Kδ in CD8^+^ T cells, we next transferred 500 WT (CD45.2) or p110δ^D910A^ (CD45.1) OT1 cells into WT (CD45.2 or CD45.1) recipients and infected them with Lm-OVA. Similar to endogenous p110δ^D910A^ CD8^+^ responses (Fig. 2), transferred p110δ^D910A^ OT1 cells generated reduced primary and secondary responses compared with transferred WT OT1 cells (Fig. 6A). After a tertiary challenge, the reduction in magnitude of the p110δ^D910A^ Tet^+^CD8^+^ T cells response was most evident in the blood and spleen (Fig. 6D). We considered whether WT OT1 T cells could outcompete p110δ^D910A^ OT1 T cells when coinjected into the same host. Contrary to our expectations, we observed equivalent expansion of p110δ^D910A^ OT1 and WT OT1 T cells both during the primary and secondary responses (Fig. 6B, 6E). However, when compared directly with coinjected WT T cells, p110δ^D910A^ T cells showed enhanced accumulation in the lymph nodes and bone marrow, which are organs associated with long-term memory (Fig. 6E). Thus, p110δ^D910A^ CD8^+^ T cell populations mediate defective responses to infection, but components of this defect are rescued by provision of WT OT1 T cells. These findings suggest a role for PI3Kδ in mediating signals required for CD8^+^ T cells to support the proliferation of other responding cells.

### PI3Kδ activity extrinsic to the responding CD8^+^ T cells also contributes to their optimal expansion

The ability of coinjected WT OT1 T cells to provide help for the p110δ^D910A^ T cells raised the question of whether other host cells also support Ag-specific CD8^+^ T cell expansion in a PI3Kδ-dependent manner. To address this question, we injected WT OT1 T cells into either WT or p110δ^D910A^ hosts (Fig. 6C, 6F). When the WT OT1 cells were injected into p110δ^D910A^ hosts, they showed reduced primary, but normal secondary, immune responses. These data indicate that factors produced by the host environment in a PI3Kδ-dependent manner are required to support the initial activation of CD8^+^ T cells. In particular, the p110δ^D910A^ host lymph nodes and bone marrow appeared deficient when it came to supporting the expansion of the transferred T cells (Fig. 6F). Nevertheless, WT OT1 T cells transferred into a p110δ^D910A^ mouse raised a secondary response of similar magnitude to those injected into a WT host (Fig. 6C). This observation indicates that whatever factor is lacking in p110δ^D910A^ hosts is not required by Tmem.

The magnitude of the CD8^+^ T cell response is dependent on effective early in vivo Ag presentation, which in turn depends on bacterial load, as treatment of mice with antibiotics 1–2 d postinfection with *L. monocytogenes* resulted in a reduced CD8^+^ T cell response (45, 46). Taking into account that p110δ^D910A^ mice clear *L. monocytogenes* more effectively than do WT mice (Fig. 4), we considered that the reduced WT CD8^+^ T cell response in a p110δ^D910A^ host might be a consequence of reduced antigenic load. To test this, we analyzed the expansion of Ag-specific CD8^+^ T cells using nonreplicating Ag by transferring WT OT1 T cells into WT or p110δ^D910A^ hosts and immunized the mice with OVA peptide and the TLR2 ligand Pam3CSK4 as an adjuvant (47, 48). p110δ^D910A^ mice immunized with OVA and Pam3CSK4 supported much lower proliferation of WT OT1 cells than did WT hosts (Supplemental Fig. 2A). Similar results were obtained when mice were immunized with OVA and LPS (a component of the cell wall of Gram^−^ bacteria) (Supplemental Fig. 2B). These results demonstrate that there are PI3Kδ-dependent host factors that are independent of effects on bacterial replication and Ag processing that promote WT CD8^+^ T cell expansion. The expression of Cxcr3, also known as CD183, favors the differentiation of Teff over Tmem (49). Supplemental Fig. 2C shows that Cxcr3 expression was reduced on Tet^+^CD8^+^ T cells isolated from blood, spleen, lymph node, and bone marrow of p110δ^D910A^ mice infected with Lm-OVA 8 d previously. However, the expression of Cxcr3 did not require PI3Kδ activity within T cells because Cxcr3 expression on WT and p110δ^D910A^ OT1 cells transferred separately (Supplemental Fig. 2D) or together (Supplemental Fig. 2E) into WT hosts was similar. In contrast, when WT OT1 T cells were transferred into p110δ^D910A^ hosts, Cxcr3 expression was reduced (Supplemental Fig. 2F). These results further demonstrate the lack of paracrine support for the differentiation of effector CD8^+^ T cells in p110δ^D910A^ mice.

### p110δ^D910A^ T cells responding to *Listeria* infection express normal amounts of cytokines and GzmB and do not show reduced DNA synthesis or increased death

**FIGURE 7.**
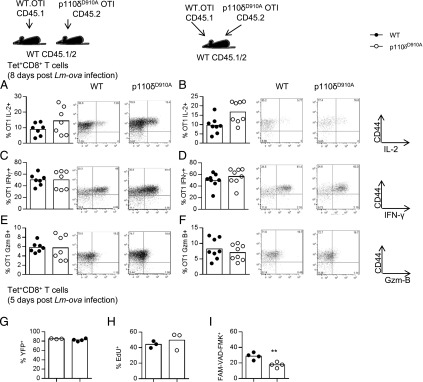
T cell cytokine and granzyme expression is not dependent on p110δ in vivo. (**A**–**F**) Five hundred WT or p110δ^D910A^ OT1 T cells were transferred to WT hosts. Mice were infected the next day with Lm-OVA and killed on day 8 postinfection. Proportion of CD44^high^ transferred cells in the spleen producing IL-2 (A and B), IFN-γ (C and D), or GzmB (E and F) from mice in which the OT1 cells were transferred into different hosts (A, C, and E) or cotransferred into the same host (B, D, and F). Representative plots are shown to the *right* of the graphs; *n* = 5–8/group. No statistically significant differences were found by an unpaired *t* test. Results are representative of two independent experiments. (**G**) WT or p110δ^D910A^ mice on the YETI background were infected with Lm-OVA. Tet^+^CD8^+^ T cells harvested from the spleen were analyzed for YFP fluorescence 5 d later. (**H**) Incorporation of the EdU by Tet^+^CD8^+^ T cells 5 d postinfection. (**I**) Staining with the caspase-binding peptide FAM-VAD-FMK by Tet^+^CD8^+^ T cells isolated from spleens 5 d postinfection. **p < 0.01.

A previous study showed that CD8^+^ T cells are themselves an important source of IL-2 production required for their optimal response to *L. monocytogenes* infections (50). The percentage of p110δ^D910A^ OT1 T cells that produced IL-2 was similar to that seen with WT OT1 cells, regardless of whether the WT and p110δ^D910A^ OT1 T cells were injected into separate or into the same WT host (Fig. 7A, 7B). To our surprise, a similar proportion of p110δ^D910A^ OT1 T cells also produced IFN-γ and GzmB (Fig. 7C–F). The in vitro differentiation assays suggested that IFN-γ production by p110δ^D910A^ T cells might be delayed rather than completely impaired. We therefore monitored IFN-γ production by Tet^+^CD8^+^ T cells 5 d postinfection, which is the earliest a distinct population of OVA-specific T cells could be readily identified. Lm-OVA–infected YETI mice revealed that a similar proportion of WT and p110δ^D910A^ Tet^+^CD8^+^ T cells produced IFN-γ also at this earlier time point (Fig. 7G). We also monitored the rate of cell division by measuring the uptake of the nucleotide analog EdU. Surprisingly, we found similar or slightly increased proportions of Tet^+^CD8^+^ p110δ^D910A^ T cells that incorporated EdU (Fig. 7H) (even though the numbers of Tet^+^CD8^+^ p110δ^D910A^ T cells recovered were lower). Therefore, the reduced number of Tet^+^CD8^+^ p110δ^D910A^ T cells does not likely reflect an inability to undergo cell division following antigenic stimulation in vivo. As an alternative explanation for the reduced magnitude of p110δ^D910A^ T cell expansion, we also considered that the loss of PI3K might increase the proportion of cells that undergo apoptosis. To test this, we measured the incorporation of the fluorescently labeled peptide FAM-VAD-FMK, which binds specifically to activated caspases by WT and p110δ^D910A^ Tet^+^CD8^+^ cells isolated from mice infected 5 d previously (Fig 7I). However, a lower proportion of p110δ^D910A^ Tet^+^CD8^+^ T cells than WT T cells showed signs of undergoing apoptosis. Hence, the requirement for PI3Kδ by CD8^+^ T cells activated in vitro to produce IFN-γ and GzmB is not shared with p110δ^D910A^ CD8^+^ T cells that respond to *L. monocytogenes* infection in vivo. Moreover, the CD8^+^ T cells that are detected several days after infection proliferate normally and do not show increased susceptibility to apoptosis. These data suggest that the signaling lesion that causes impaired expansion of p110δ^D910A^ CD8^+^ T cells may manifest itself relatively early during the immune response and provide evidence that redundant pathways cooperate with PI3Kδ to promote cytokine expression and cytolytic function in response to infection in vivo.

## Discussion

In this study, we have shown that PI3Kδ controls the magnitude of both primary and secondary CD8^+^ T cell responses in response to infection with the intracellular bacterial pathogen *L. monocytogenes*. However, inhibition of PI3Kδ does not prevent the differentiation of naive CD8^+^ T cells into competent long-lived CD8^+^ Tmem, nor for Teff to produce IFN-γ and GzmB in response to infection in vivo. The ability of CD8^+^ T cells to generate a full-magnitude primary response to *L. monocytogenes* infection depends in part on PI3Kδ activity within responding T cell populations.

Recent work form Hodgkin and colleagues (51) has demonstrated that CD8^+^ T cells integrate signals from the Ag receptor, costimulatory receptors, and cytokine receptors in a linear manner to determine their “division destiny,” which represents the total number of divisions each cell will undergo before contraction of the immune response. We propose that transduction of antigenic signals is weaker in p110δ^D910A^ T cells, and this has long-term consequences in terms of the magnitude of clonal expansion by limiting the number of CD8^+^ T cells that will reach their full proliferative capacity, even though a snapshot of T cells responding to the Ag (several days after infection) revealed unperturbed proliferation. Possible mechanisms that might explain such reduced proliferative capacity after initial activation may include fewer cells that respond to *L. monocytogenes*, delayed or sustained first divisions, or selective loss of daughter cells after the first division. We are currently analyzing the division of transferred T cells during the first 24–48 h postinfection to evaluate these possibilities. It is also possible that aberrant expression of homing receptors such as CD62L and Cxcr3 prevents p110δ^D910A^ T cells from fully engaging with the relevant APCs during the early stages of their response to infection. However, the reduced number of Tet^+^CD8^+^ Teff did not appear to be caused by altered anatomical redistribution, as we did not find any organ with increased numbers of endogenous Tet^+^CD8^+^ T cells in p110δ^D910A^ mice relative to WT mice infected with *L. monocytogenes*.

When we only injected 100 OT1 T cells, we observed comparable expansion of T cells from WT and p110δ^D910A^ donors. Similarly, we also demonstrated in OT1 cotransfer experiments that Ag-specific WT CD8^+^ T cells can provide help to other responding CD8^+^ T cells such that the p110δ^D910A^ OT1 T cells expand normally under conditions of cotransfer. Presumably, in both of these cases, there will be an excess of endogenous and/or cotransferred WT OVA-specific T cells also responding to Lm-OVA. Our data and previously published data suggest an endogenous pool of SIINFEKL-specific CD8^+^ T cells equivalent to 500 transferred OT1 cells. Hence endogenous SIINFEKL-specific CD8^+^ T cells would exceed transferred p110δ^D910A^ OT1 cells by a ratio of 5:1 when 100 cells were injected. Similar calculations estimate the ratio of WT to p110δ^D910A^ to be 3:1 in the cotransfer experiments (500 WT endogenous Tet^+^CD8^+^ cells and 250 cotransferred WT OT1 cells against 250 p110δ^D910A^ OT1 cells). It seems, therefore, that the WT Ag-specific CD8^+^ T cells need to outnumber the p110δ^D910A^ T cells by a factor of ∼3:1 or more to provide sufficient signals to support normal expansion of the p110δ^D910A^ OT1 T cells. We also consider the alternative hypothesis that p110δ^D910A^ OT1 cells may impede the expansion of cotransferred WT OT1 cells. However, when larger numbers of p110δ^D910A^ T cells were injected into WT hosts, they did not appear to be more potent than WT OT1 cells at suppressing endogenous responses (which would be expected if p110δ^D910A^ T cells suppressed the expansion of WT T cells). Hence, we favor the hypothesis that CD8^+^ T cells responding to the same Ag can support one another to promote optimal proliferation and that their ability to provide such help depends on PI3Kδ.

In addition to the CD8^+^ T cells themselves, it is likely that PI3Kδ expressed by other cell types also contributes to the production of cytokines and/or costimulatory ligands that promote CD8^+^ T cell expansion. Thus, when WT OT1 cells were transferred into a p110δ^D910A^ host, the T cells showed impaired expansion and differentiation when compared with a WT host. We have previously shown that the deletion of Pten within activated CD4^+^ T cells leads to enhanced Tet^+^CD8^+^ T cell responses to Lm-OVA infection (52). CD4 T cells express a number of ligands and cytokines that help facilitate optimal CD8^+^ T cell responses, and PI3Kδ-deficient T cells produce less IL-2, IFN-γ, IL-21, and CD40L (6, 53–55). It is also possible that PI3Kδ-deficient innate immune cells fail to support optimal CD8^+^ T cell expansion.

Our studies showed that whereas the Teff response was diminished in p110δ^D910A^ mice, the production and maintenance of Tmem were largely intact. Administration of a low dose of rapamycin has been shown to enhance the generation of memory T cells following infection (23). Whether the level of inhibition of mTOR in those studies was comparable to that observed when PI3Kδ is inactivated remains to be determined. It is nevertheless interesting that in addition to promoting Tmem, rapamycin is the only drug that has been proven to delay age-related decline in mice (56). We have previously shown that genetic alterations that prolong life by altering PI3K and/or mTOR signaling also preserve the balance of naive T cells to Tmem (which is higher in young mice than in old mice) (57, 58). In this context, it is pertinent to note that although p110δ^D910A^ mice mounted reduced magnitude CD8^+^ T cell responses, old p110δ^D910A^ mice did not show as strong evidence of age-related decline as displayed by the WT mice. Further studies are needed to establish whether PI3Kδ inhibition can delay aging-related decline of the immune system.

The results presented in the present study have potential clinical implications. The PI3Kδ inhibitor idelalisib is now an approved drug for the treatment of chronic lymphocytic leukemia (59, 60). So far, there is little evidence that PI3Kδ inhibitors increase the risk of infection in these patient populations. The results presented in the present study suggest that, at least in some contexts, PI3Kδ inhibition may in fact help limit bacterial infection. Furthermore, our findings raise the possibility that PI3Kδ inhibitors could be used to augment immune function in the context of certain acute and chronic viral and bacterial infections.

We have recently shown that p110δ^D910A^ mice are resistant to cancer and that this resistance is dependent on CD8^+^ T cells (33). Moreover, p110δ^D910A^ mice that rejected an initial tumor inoculation were highly resistant to a second challenge with a higher tumor dose, suggesting intact formation of CD8^+^ Tmem also in the context of cancer (33). However, the resistance to cancer was more striking in mice in which p110δ was only deleted in regulatory T cells, indicating that PI3Kδ inhibition did have some adverse effects on effector cells, possibly including CD8^+^ T cells (33). In this context, it is interesting that delayed inhibition of PI3Kδ (drug added 24 after activation) inhibited CD4^+^ T cells, but less so CD8^+^ T cells (6). Moreover, the inhibition of PI3Kδ within differentiated CD8^+^ T cells has minimal effect on the ability of CTLs to kill cancer cells (33, 61). The present study may thus form the basis for designing strategies that will favor more robust CD8^+^ T cell responses while maintaining suppression of regulatory T cell function in the context of PI3Kδ inhibition. Indeed, tumor vaccines, which can include *L. monocytogenes*, may be one such approach (62).

Activated PI3Kδ syndrome patients that harbor activating mutations in the gene that encodes p110δ (PIK3CD) suffer from recurrent bacterial and viral infections (63, 64). Interestingly, the patients have abundant activated or exhausted CD8^+^ cells and lack CD8^+^ Tmem. In these patients, constitutive PI3Kδ signaling may thus have favored the differentiation of CD8^+^ Teff over Tmem, in direct contrast to the p110δ^D910A^ mice in which CD8^+^ Tmem persist, but CD8^+^ Teff are less abundant. It will be of interest to determine whether PI3Kδ inhibitors can restore normal CD8^+^ T cell function in activated PI3Kδ syndrome patients.

In summary, PI3Kδ inhibition attenuates immediate CD8^+^ Teff responses, but it is not detrimental to the generation of long-lived CD8^+^ Tmem responses. These observations should help inform therapeutic use of PI3Kδ inhibitors for diverse clinical indications.

## Supplementary Material

Data Supplement
