# Supplementary material for: PI3Kδ Regulates the Magnitude of CD8+ T Cell Responses after Challenge with Listeria monocytogenes
Source: J Immunol. 2015 Aug 26;195(7):3206–17. doi: 10.4049/jimmunol.1501227 (PMC4574522; doi:10.4049/jimmunol.1501227)
Supplement: Data Supplement [file JI_1501227.zip › JI_1501227_Supplemental_Figures_1.pdf]

Supplemental Figure 1. CD62L and CCR7 expression by Tet⁺CD8⁺ T cells in the blood at day 8 post infection

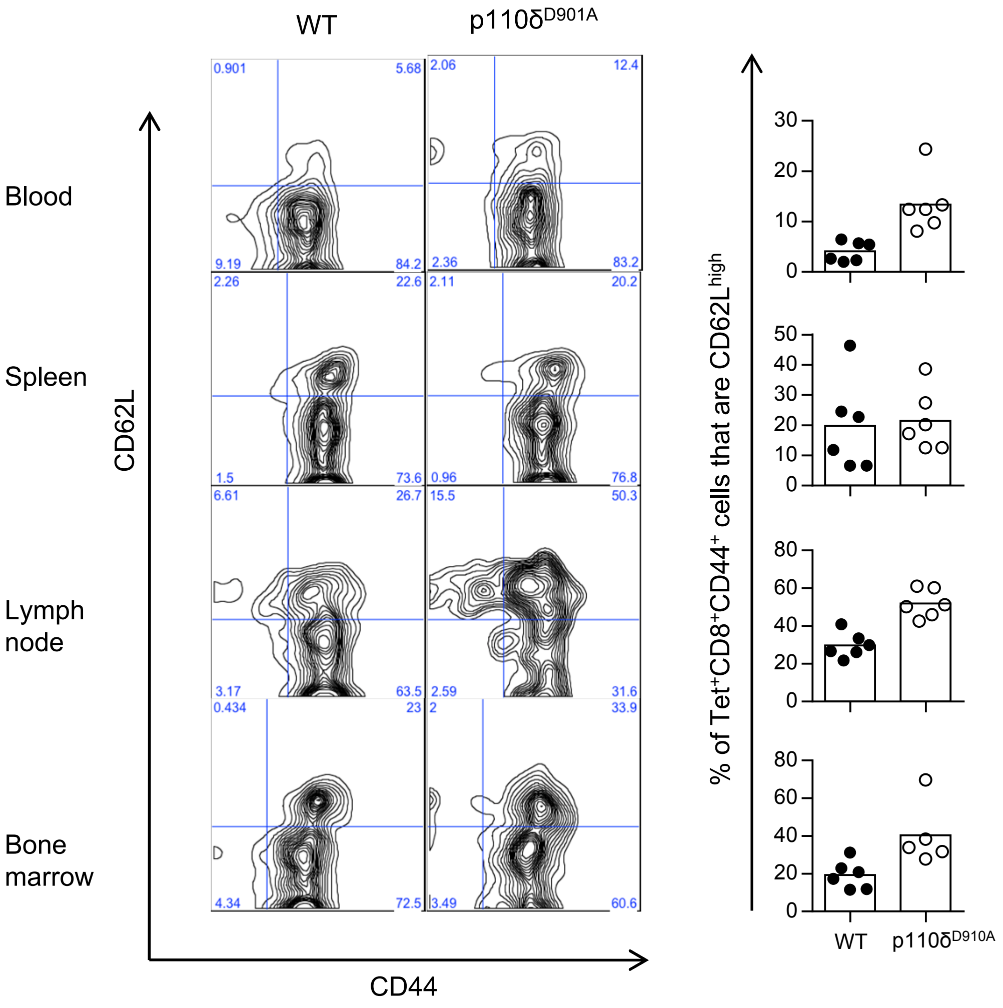

CD44 and CD62L expression by Tet$^+$CD8$^+$ T cells in the blood at day 8 post infection. Representative of two independent experiments.

Supplemental Figure 2. T cell response to non-replicating antigens and Cxcr3 expression

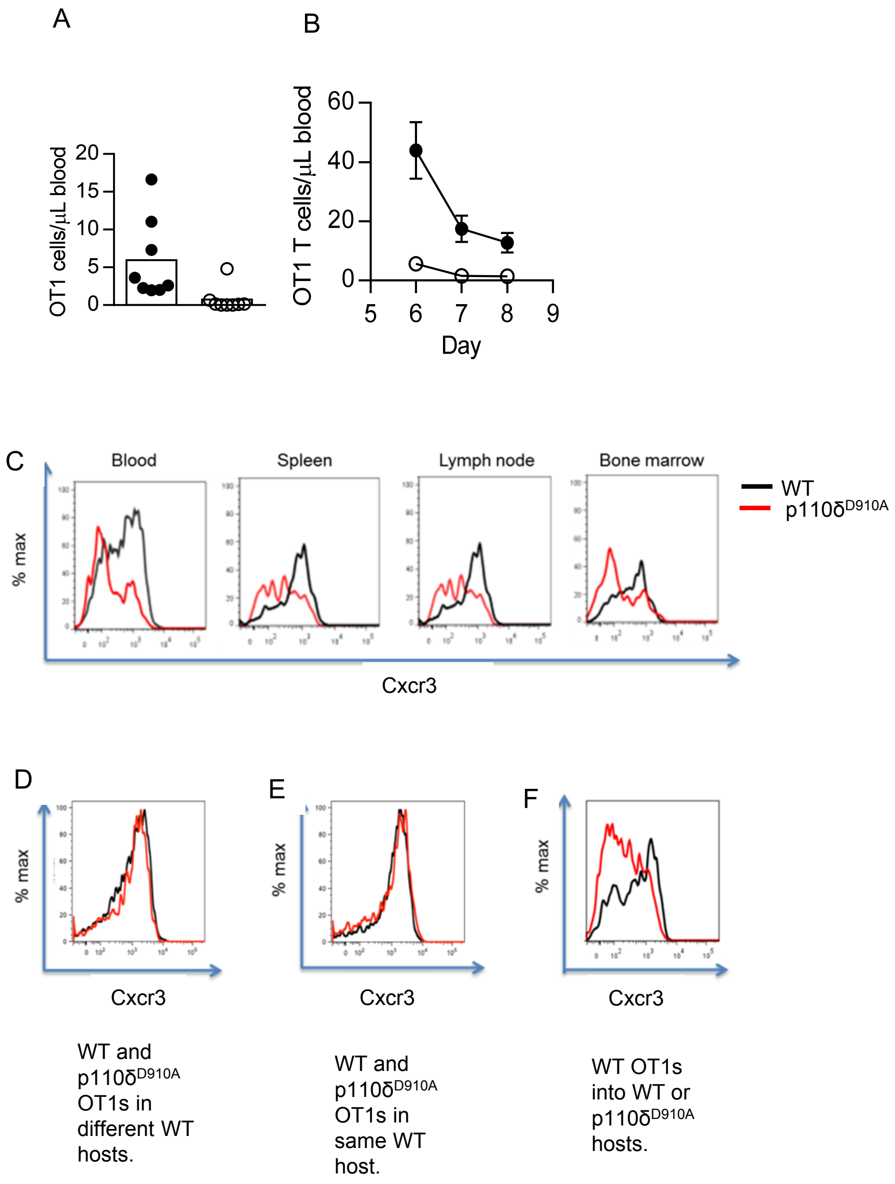

A, B. 500 OT1 cells were transferred into WT or p110δ^{D910A} hosts which were immunized with SIINFEKL peptide resuspended in Pam4CK (A) or Ova protein resuspended in LPS (B). The number of OT1 cells per ul blood is shown. **A.** OT1 cells detected 5 days after immunization. **B.** OT1 cells detected 6-8 days after immunization.
C. Cxcr3 expression on Tet^{+}CD8^{+} T cells isolated from WT (black line) or p110δ^{D910A} (red line) mice that had been infected with *Lm*-ova 8 days previously.
D, E. Cxcr3 expression on WT (black) or p110δ^{D910A} (red) OT1 cells that had been transferred into different (D) or the same (E) WT hosts which were then infected with *Lm*-ova. The OT1 T cells were recovered 8 days later.
F. WT OT1 cells that had been transferred into WT (black) or p110δ^{D910A} (red) host and recovered 8 days after infection with *Lm*-ova.
